# Dental caries experience and associated factors in adults: a cross-sectional community survey within Ethiopia

**DOI:** 10.1186/s12889-021-10199-9

**Published:** 2021-01-21

**Authors:** Birke Bogale, Fasikawit Engida, Charlotte Hanlon, Martin J. Prince, Jennifer E. Gallagher

**Affiliations:** 1 Faculty of Dentistry, Oral and Craniofacial Sciences, Centre for Host Microbiome Interactions, King’s College London, London, UK; 2 Department of Dental and Maxillofacial Surgery, St Paul’s Hospital Millennium Medical College, Addis Ababa, Ethiopia; 3 Department of Dentistry, College of Health Sciences, Addis Ababa University, Addis Ababa, Ethiopia; 4 Institute of Psychiatry, Psychology and Neuroscience, Health Service and Population Research Department, Centre for Global Mental Health, King’s College London, London, UK; 5 College of Health Sciences, School of Medicine, Department of Psychiatry, WHO Collaborating Centre for Mental Health Research and Capacity-Building, Addis Ababa University, Addis Ababa, Ethiopia; 6 Centre for Innovative Drug Development and Therapeutic Trials for Africa (CDT-Africa), College of Health Sciences, Addis Ababa University, Addis Ababa, Ethiopia

**Keywords:** Dental caries, Dental caries experience, Oral health, Health system, Ethiopia

## Abstract

**Background:**

Ethiopia is a developing sub-Saharan African country with increasing prevalence of non-communicable diseases (NCDs), including oral conditions. Oral health and dental care have been given little consideration, and there is limited information relating to population oral health and use of dental services in the country. The aim of this study was to examine the burden and associated factors of dental caries experience and investigate access to dental care amongst adults within Ethiopia.

**Methods:**

This community-based oral health survey is a baseline study for the ASSET - Health System Strengthening in sub-Saharan Africa project undertaken in the Butajira area, south-central Ethiopia. A stratified random sample of households and individuals participated in the study. The survey instruments were mainly based on the WHO Oral Health Survey Methods manual (5^th^ ed.). Face-to-face interviews and clinical dental examinations were conducted. The data were analysed for descriptive statistics; and Poisson regression models were built to assess the association of dental caries and predictor variables in adults (≥18 years).

**Results:**

Most of the study population (*n* = 626) were female (63.9%), married (71.4%) and Muslim (76.0%). Just over half (53.2%) lived in rural areas and many (44.4%) had no formal education. A majority (74.0%) reported never utilising dental care services, and the main reason was never experiencing any dental problem (71.3%). Sixty percent (*n* = 377) of the adults had experienced dental caries, 88.0% (*n* = 332) of whom had untreated carious teeth. Pain or discomfort was reported by 16.5, and 7.2% had one or more PUFA component. Most (59.9%) adults with dental caries experience reported tooth pain or discomfort during the last year. In the fully adjusted Poisson regression model, increasing age, dental care utilisation and Khat chewing had positive significant associations with dental caries experience, whilst education status was negatively associated (*p* < 0.05).

**Conclusion:**

This study demonstrated a high burden of dental caries and considerable consequences resulting from untreated disease in this population of adults. There was evidence of social inequity, limited utilisation of dental care and oral health awareness. This highlights the need for oral health system strengthening focusing on health promotion and expanding overall access to care.

**Supplementary Information:**

The online version contains supplementary material available at 10.1186/s12889-021-10199-9.

## Background

Oral health is an integral element of good health and wellbeing as it facilitates essential functions, most notably: eating, speaking, smiling, and socialising [1]. Oral conditions, especially dental caries and periodontal diseases are considered major global public health problems [2–4]. Although mostly preventable and treatable, they can be life threatening. About 90% of the world population face at least one form of oral condition sometime in their lives and oral conditions have been associated with adverse socioeconomic impact in both adults and children irrespective of their gender [5].

Oral health in Africa has been considered a low priority in relation to communicable diseases, and reliable data are scarce. However, non-communicable diseases (NCDs), including oral conditions are becoming more prevalent; and there are substantial oral health inequalities in both high- and low-income nations across the continent including Ethiopia [6, 7]. This is mainly related to specific risk factors associated with nutritional transitions - transition towards the ‘Western diets’ involving increased sugar consumption, along with other factors [8].

Ethiopia is a large and diverse developing sub-Saharan African country in the east African region. It has one of the world’s fastest growing economies with most of the population living in rural areas. Considerable economic and health inequalities exist between the urban and rural populations nationally [9, 10]. Ethiopian oral health data are notably scarce, and there are no community-based oral health surveys. However, local hospital and school-based cross-sectional studies suggest untreated dental caries present a problem. First, in two local studies in north-western Ethiopia, untreated dental caries was identified in about one-third and one-fifth of schoolchildren aged 7–14 and 6–15 years, respectively. The disease was positively associated with toothache, and negatively associated with oral hygiene, household income and paternal education [11, 12]. Second, findings from hospital attendees, involving children and adults, indicate that dental caries, which affected three-quarters of attendees, was the most frequent reason for presentation at the dental service [13, 14]. Healthcare in Ethiopia is improving [15]. However, access to health services in general is still harder for the rural population [16, 17], and the dental services in particular are very limited. It is predominantly found in the private sector as well as in secondary and tertiary referral public hospitals which are largely located in the cities and bigger towns [18, 19].

Africa possesses only 1% of the global dental workforce [20, 21], and has a dentist: population ratio of around 1: 40,000. Ethiopia’s dentist-to-population ratio has historically been extremely low at 1:1.5 million population. Besides, inter-regional disparities are common, particularly between the urban and rural areas [20]. In addition, as with other sub-Saharan African countries, Ethiopia loses its high-end healthcare professionals due to migration to more developed countries, and the supply does not match the demand because of the low retention [22, 23]. This, together with the wider social determinants [24], such as individual lifestyle, community influences, working and living conditions; and the more general social conditions contribute to the volume of untreated disease including oral conditions in the country [3, 25]. Since oral conditions are mostly associated with reversible or modifiable risk-factors, and most are preventable, their magnitude and late complications can be controlled. This can be addressed by appropriate public health measures, and oral health policy and strategy set at the government level. For this to happen, up-to-date profile of the diseases: normative and perceived data, along with their determinants should be known.

The ‘Health System Strengthening in sub-Saharan Africa – ASSET’ project in Ethiopia is led by King’s College London within the National Institute for Health Research (NIHR) Global Health Research grant. It is a collaborative project with Addis Ababa University, and it provides an opportunity to support the country’s healthcare system through health system strengthening interventions across its three phases: diagnostic, piloting and implementation. The aim of the dental component within the diagnostic phase of the surgical care survey was to examine the burden of oral disease and explore access to dental care and its possible barriers in a rural and urban population within Ethiopia to inform health system strengthening. This will be facilitated using the baseline information to inform the second and third phases of the ASSET project successively.

In light of the above, the aim of this paper is to report the burden and associated factors of dental caries experience and to investigate access to dental care amongst adults within Ethiopia.

## Methods

This community based oral health survey was part of the surgical care survey of the ASSET project in Ethiopia. Whilst we collected epidemiological data from children and adults using standardised questionnaires and clinical oral examinations from March 1–30, 2019, this paper includes data from adults only.

### Study area and population

The study was conducted in districts across the Butajira Health and Demographic Surveillance Site (HDSS), south-central Ethiopia. The Butajira HDSS is found in Meskan and Mareko districts in the Gurage zone, Southern Nations, Nationalities, and Peoples’ Region (SNNPR) of Ethiopia. It covers nine rural and one urban sites, with a total of 40,000 people and an area of approximately 797 km^2^, of which 9 km^2^ is Butajira town - the only urban site of the HDSS. Butajira is located 130 km south of the capital, Addis Ababa. The main source of income for the rural population in the HDSS is farming. The production mainly includes Teff (a cereal staple), barley, millet, legumes and maize on the rich soil of the area, with the staple food of Enset (false banana). Butajira also grows coffee, chilli peppers and Khat (a mild stimulant drug) as cash crops [26]. This area was chosen for the project as it has an established rural health programme and it provides a study base for essential health research and intervention.

The sample size employed for the dental survey was calculated for the ASSET surgical community survey in order to detect a prevalence of unmet need for surgical care in the community; and it was 1200: 2 persons of any age, randomly selected from 600 households. The sample population was stratified across the seven accessible sub-districts (*kebeles*) of Butajira town and Meskan district (*woreda*). Civil unrest prevented access to the remaining HDSS sites. The households were randomly selected with probability proportionate to the size of the total population in the sub-districts. Random sampling was again used to select two persons within the households using the HDSS sampling frame.

Participants included in this study were those living in Butajira HDSS for at least 1 year prior to the survey and able to communicate in the working language, Amharic. The adult participants were those willing to participate in the study and provide written consent. People with serious health conditions, making it difficult to comply with the interview and oral examination, were excluded from the study.

### Instruments and measures

The 5th edition of World Health Organisation (WHO) Oral Health Survey Methods manual (5^th^ ed.) [27], formed the basis for the survey methodology. Some elements in the data collection instruments (Oral Health Questionnaire – ‘Additional file 1’, and Oral Health Assessment form for Adults) were adapted from the UK (England, Wales and Northern Ireland) adult dental health survey 2009 methodology [28], and the dental caries threshold was determined based on the International Caries Detection and Assessment System (ICDAS) criteria [29], within the WHO dentition status assessment format [27].

Dental caries experience at tooth level, and the consequences of untreated caries were the main dependent variables. Dental caries experience was determined using the DMFT index (the presence of Decayed, Missed and Filled Teeth) extracted from the ‘dentition status’ data based on the WHO oral health survey guideline (page 42) [27]. The threshold of individual carious lesions was determined using the International Caries Classification and Management System (ICCMS) ICDAS coding criteria. We considered ICDAS 4 (D_4_), and above as ‘caries’, because ICDAS 3 (D_3_) and below is more difficult to assess when only visual examination is used without clinical/diagnostic facilities [29]. This is particularly more difficult to identify early caries in areas where Khat chewing is common, as it can stain teeth after repeated use. Khat, a green leaf chewed for its mild stimulating effect, is commonly used in East-Africa and Middle-east region [30, 31].

To determine the consequences of untreated caries, we used the PUFA index (the presence of Pulpal involvement, Ulcer, Fistula and Abscess) [32]. We also assessed the presence of pain (toothache) or discomfort as an additional outcome variable [28].

Sociodemographic factors included as important independent variables for subsequent analysis were: age, sex, education status, religious affiliation, marital status and location (urban/rural) as these variables are common predictors of caries [33, 34]. They were extracted from the baseline surgical community survey questionnaire instrument. In relation to lifestyle and behavioural factors, frequency of tooth cleaning and materials used, including toothpaste were collected in the dental data collection instrument and used as proxies for oral hygiene/plaque removal and fluoride exposure [35, 36]. As per the situational analysis prior to the study, other mechanisms of fluoride exposure in the community is not known, apart from toothpastes as those widely available in the market contain fluoride. Other health behavioural and lifestyle factors examined because of their influence on oral health were: Khat chewing, sugar, tobacco and alcohol consumption [8, 37]. Self-reported utilisation of dental care, which refers to attending a recognised dental service provider was also obtained to look at access to dental service and possible barriers to care [13, 38].

### Standardization

The dental questionnaire and clinical data collection form adapted and utilised in this study was a standardized oral health survey data collection instrument. The questionnaire was translated into the working language of the area – Amharic. It was then back translated into English by two native Amharic speakers with full professional competencies in English. They were non-dental/medical professionals with no knowledge about the survey. The original and translated questionnaires were then assessed if they achieve semantic and conceptual equivalence, and they did not require any further amendment.

The dental data collectors (dentists) were trained over a two-day period and calibrated for identification and assessment of dental caries. This training was made online via skype using power point and interactive presentations, and it was aided by the ICCMS ICDAS training website. During the training, extracted teeth were used for identification of dental caries based on the threshold set for the purpose of this study. The dental study team at King’s College London trained the dental data collectors, and they were calibrated against our gold standard examiner (ICDAS expert). To assess the reliability of dental caries data collection, inter-examiner agreement was computed using kappa statistics. The kappa value in our analysis ranged from 0.6–1.0 (mean: 0.96) which allowed us to proceed with the data collection. A standard form was created online for recording of the data. Repeat examinations were not possible because of time and logistical constraints.

### Data collection

Lay data transcribers and dentists teamed up with their surgical community survey counterparts during the data collection. The dentists interviewed the study participants face-to-face and examined them for oral diseases in their respective households. The transcribers recorded all information (questionnaire and clinical oral examination) using the Open Data Kit (ODK) software on smart phones. Oral examination was visual, conducted using standard disposable dental mirrors and blue-white spectrum solar headtorches with the examinee supine. They were lying on household benches. Dental probes were only used for removing debris, when required.

### Data entry, cleaning and analysis

The ASSET data specialist exported the data and merged the surgical and dental data using unique identifier codes to form the datasets for analysis. We then cleaned the dental data removing any incomplete cases related to this analysis, created new variables, and undertook the data analysis using SPSS version 25 and STATA version 16. The numerical variable – age was recoded into six age-bands [27], and the variable dental caries experience was categorized in to D_4_MFT = 0 and D_4_MFT ≥ 1 prior to the data analysis. Sugar consumption ‘risk’ was also calculated based on the frequency of reported intake of sweets, cakes, biscuits, and soft/fizzy drinks. Consuming at least one of them more than once a week was categorised as ‘higher-risk’, between once a month and once a week as ‘moderate-risk’, and rarely or never as ‘lower-risk’ consumption. Since the reported sugar consumption was very low, this categorisation was only made conventionally.

The sociodemographic characteristics, health behaviours and other relevant data were summarized using descriptive statistics. We used frequencies and proportions to describe the variables and used a chi-square test to examine the association between the independent and dependent variables outlined above. The association of health behaviours, tooth cleaning habit and sociodemographic factors with dental caries experience were further analysed using Poisson regression models, and prevalence ratios with 95% confidence intervals were reported.

## Results

Of the 1071 dental survey participants (89.3% response rate), 650 were adults. Twenty-four adult participants (3.8%) were excluded from the final analysis because of missing relevant information. Therefore 626 participants, with an average age of 38.4 years (range: 18–100; sd: 16.4 years) were included for analysis. A higher proportion of the study population was female (63.9%, *n* = 400), married (71.4%, *n* = 447), and Muslim (76%, *n* = 476). Just over half (53.2%, *n* = 333) lived in rural areas and 44.4% (*n* = 278) had not received any formal education.

Reported health behaviours were largely positive. The vast majority (97.9%, *n* = 613) reported never smoking cigarettes or drinking alcohol (90.4%, *n* = 566). A majority reported not chewing Khat (59.0%, *n* = 370); whilst sugar consumption was generally low with only 10.7% (*n* = 67) in the ‘higher-risk’ category (consuming any sweet more than once a week). However, tooth cleaning twice a day or more was only practiced by about one in five adults, as was toothpaste use. Mefakiya (Chewsticks) were commonly used for tooth cleaning (45.0%, *n* = 281).

A majority (74.0%, *n* = 463) of participants had never utilised dental care services. The main reported reason for not utilising a dental service was never experiencing any dental problem (71.3%, *n* = 330), followed by the distance to a dental clinic/service (9.3%, *n* = 58) and the cost of treatment (8.2%, *n* = 51). Only 37.1% (*n* = 140) of those with dental caries experience reported utilising a dental service.

Dental caries experience D_4_MFT and its consequences in relation to sociodemographic and behavioural variables are presented in Tables 1 and 2. Six out of 10 (60.2%, *n* = 337) of adults surveyed had a D_4_MFT score of 1 or more, out of which 88.0% (*n* = 332) had at least one untreated.

**Table 1:** Bivariate analysis of dental caries experience and consequences of untreated dental caries (D_4_MFT ≥ 1)^a^ with sociodemographic factors of adults in Butajira HDSS, south-central Ethiopia

Factors		Total		DT ≥ 1			MT ≥ 1			DMFT ≥ 1			Pain/Discomfort			PUFA ≥ 1		
		n	%	n	%	***p***-value	n	%	***p***-value	n	%	***p***-value	n	%	***p***-value	n	%	***p***-value
**Total**		**626**	**100**	**332**	**53.0**		**188**	**30**		**377**	**60.2**		**103**	**16.5**		**45**	**7.2**	
**Age group**	18–24	123	19.7	35	28.5	**0.001**	5	4.0	**0.001**	36	29.3	**0.001**	**8**	6.5	**0.001**	**7**	5.7	0.203
	25–34	190	30.4	70	36.8		39	20.5		85	44.7		22	11.6		13	6.8	
	35–44	103	16.5	65	63.1		30	29.1		78	75.7		17	16.5		3	2.9	
	45–54	79	12.6	56	54.4		36	35.0		61	59.2		18	22.8		8	10.1	
	55–64	74	11.8	59	79.7		40	54.0		64	86.5		26	35.1		9	12.2	
	65+	57	9.1	47	82.5		38	66.7		53	93.0		12	21.1		5	8.8	
**Sex**	Female	400	63.9	213	53.3	0.886	126	31.5	0.165	240	60.0	0.932	65	16.3	0.911	28	6.0	0.872
	Male	226	36.1	119	52.7		62	27.4		137	60.6		38	16.8		17	7.5	
**Education level**	No formal education	278	44.4	205	73.7	**0.001**	121	43.5	**0.001**	226	81.3	**0.001**	**65**	23.0	**0.001**	19	6.8	0.78
	Primary school	202	32.3	88	43.6		47	23.3		102	50.5		23	11.4		16	7.9	
	Secondary school	97	15.5	26	26.8		14	14.4		34	35.1		11	11.3		8	8.3	
	Higher education	49	7.8	13	26.5		6	12.2		16	32.7		4	8.16		2	4.1	
**Religion**	Muslim	476	76.0	261	54.8	0.183	149	31.3	0.135	296	62.2	0.166	76	16.0	0.810	31	6.5	0.495
	Orthodox	131	21.0	62	47.3		37	28.2		72	55.0		24	18.3		12	9.2	
	Protestant	19	3.0	8	42.1		2	10.5		9	47.4		3	15.8		2	10.5	
**Marital Status**	Single/ Never Married	106	16.9	30	28.3	**0.001**	10	33.3	**0.001**	32	30.2	**0.001**	**8**	7.6	**0.017**	2	1.9	0.068
	Married	447	71.4	242	54.1		134	55.4		278	62.2		79	17.7		37	8.3	
	Formerly Married	73	11.7	60	82.2		44	60.3		67	91.8		16	21.9		6	8.2	
**Location**	Urban	293	46.8	127	43.3	**0.001**	81	27.7	0.222	154	52.6	**0.001**	37	19.8	**0.015**	23	7.9	0.548
	Rural	333	53.2	205	61.6		107	32.1		223	66.8		66	23.0		22	6.6	

^a^ DT: ICDAS 4 and above

**Table 2:** Bivariate analysis of dental caries experience and consequences of untreated dental caries (D_4_MFT ≥ 1)^a^ with relevant health behavioural factors of adults in Butajira HDSS, south-central Ethiopia

Factors		Total		DT ≥ 1			MT ≥ 1			DMFT ≥ 1			Pain/Discomfort			PUFA ≥ 1		
		n	%	n	%	***p***-value	n	%	***p***-value	n	%	***p***-value	n	%	***p***-value	n	%	***p***-value
**Utilisation of dental care services**	Never	463	74.0	212	45.8	**0.001**	100	21.6	**0.001**	237	51.2	**0.001**	66	14.3	**0.012**	26	5.6	**0.010**
	Ever	163	26.0	120	73.6		88	54.0		140	85.9		37	22.7		19	11.7	
**Frequency of tooth cleaning**	Never/ Rarely	65	10.4	48	73.9	**0.001**	27	41.5	**0.007**	51	78.5	**0.001**	9	13.9	**0.001**	2	3.1	**0.001**
	Less than once a day	305	48.7	156	51.2		79	25.9		178	58.4		50	16.4		15	4.9	
	Once a day	135	21.6	53	39.3		35	25.9		65	48.2		15	11.1		8	5.9	
	Twice or more per day	121	19.3	75	62.0		47	38.8		83	68.6		83	68.6		20	16.5	
**Tooth cleaning material**	Mefakiya ^b^ / Chewstick	281	44.9	150	53.4	**0.001**	87	31.0	**0.004**	169	60.1	**0.001**	53	18.9	0.288	20	7.1	**0.006**
	Wooden toothpicks	142	22.7	75	52.8		45	31.7		89	62.7		22	15.5		7	4.9	
	Toothbrush	114	18.2	44	38.6		19	16.7		52	45.6		13	11.4		10	3.1	
	Other	24	3.8	15	62.5		10	41.7		16	66.7		6	25.0		6	7.1	
	Do not clean	65	10.4	48	73.9		27	41.5		51	78.5		9	13.8		2	4.9	
**Use of toothpaste**	Yes	118	18.9	42	35.6	**0.001**	17	14.4	**0.001**	50	42.4	**0.001**	14	11.9	0.260	8	8.8	0.374
	No	443	70.8	242	54.6		144	32.5		276	62.3		80	18.1		35	25.0	
	Do not clean	65	10.4	48	73.9		27	41.5		51	78.5		9	13.8		2	3.1	
**Frequency of Khat chewing**	Never Chew	370	59.1	164	44.3	**0.001**	90	24.3	**0.003**	193	52.2	**0.001**	54	14.6	0.484	23	6.2	0.519
	Less than a week	72	11.5	43	59.7		26	36.1		48	66.7		13	18.1		8	11.1	
	More than once a week	101	16.1	64	63.4		40	39.6		70	69.3		19	18.8		8	7.9	
	Every day or nearly every day	83	13.3	61	73.5		32	38.6		66	79.5		17	20.5		6	7.2	
**Level of sugar consumption risk**	Higher^c^	67	10.7	31	46.3	**0.004**	13	19.4	**0.017**	35	52.2	**0.001**	9	2.0	0.733	4	0.1	0.438
	Moderate^d^	223	35.6	102	45.7		59	26.5		115	51.6		36	0.4		20	20.0	
	Lower^e^	336	53.7	199	59.2		116	34.5		227	67.6		58	68.2		21	24.7	
**Toothache or discomfort (in past 12 months)**	Often	44	7.0	37	84.1	**0.001**	24	54.6	**0.001**	38	86.4	**0.001**	22	0.5	**0.001**	12	27.3	**0.001**
	Occasionally	133	21.3	104	78.2		58	43.6		113	85.0		50	37.6		14	10.5	
	Rarely	97	15.5	66	68.0		35	36.1		75	77.3		14	14.4		4	4.1	
	Never	352	56.2	125	35.5		71	20.2		151	42.9		16	4.6		14	4.1	

^a^DT: ICDAS 4 and above, ^b^Mefakiya: an Amharic name for chew-stick, ^c^More than once a week, ^d^Between once a month and once a week, ^e^Rarely or never

**Fig. 1:**
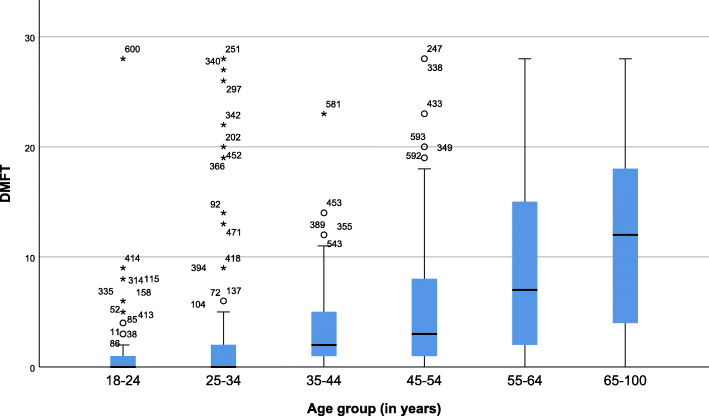
Progression of dental caries experience (DT: ICDAS 4 and above) across the age categories of adults in Butajira HDSS, South-central Ethiopia

D_4_MFT scores ranged from 0 to 28 (mean, 4.4 ± 6.9), and the score increased with age (Fig. 1). Whereas 49.9% (*n* = 188) of adults had missing teeth, only 0.8% (*n* = 3) of those with dental caries experience had fillings. Pain or discomfort was reported by 16.5% (*n* = 103) of the study participants, with 7.2% (*n* = 45) having one or more PUFA component. Pulp involvement encompassed the majority (80.0%, *n* = 36) of PUFA. Most (59.9%, *n* = 226) adults with dental caries experience reported tooth pain or discomfort during the last year.

Based on the bivariate analysis, increasing age had a significant and positive association with dental caries experience, whereas educational level was negatively associated. Marital status and location of residence also demonstrated high significant associations with caries (*p* < 0.001 for all). However, no association was observed with sex and religion. Regarding the health behaviours, dental care service utilisation, frequency of tooth cleaning and materials used (including the use of toothpaste), sugar intake and Khat chewing were highly associated with dental caries experience (*P* < 0.001 for all). There were also significant associations between the health impact, notably tooth pain or discomfort; and age, education, marital status, dental care service utilisation, location of residence and tooth cleaning frequency (*p* < 0.05). Out of all the independent variables, only dental care service utilisation, tooth cleaning frequency and materials used for dental hygiene were significantly associated with PUFA (*p* < 0.05).

The Poisson regression models for dental caries experience and predictors are presented in Table 3. In the unadjusted model, all the independent variables, except for sex, had significant associations with dental caries experience (*p* < 0.05). In the adjusted model, sociodemographic variables, age and educational status remained significantly associated. The probability of experiencing dental caries was higher in older age-groups and lower in higher levels of education. The probability of experiencing dental caries was 1.5 times (95%CI: 1.4–1.7, *p* < 0.001) higher in persons who reported utilising dental care services than in those who never did. Khat was the only lifestyle behavioural component that remained significant in the adjusted model. Persons who reported chewing Khat daily or nearly every day had 1.4 times (95%CI: 1.2–1.6, *p* < 0.001) the probability of experiencing dental caries as compared with reported non-chewers. Location of residence and most of the health behaviours including tooth cleaning and the risk of sugar intake did not predict the probability of experiencing caries after adjusting for other factors.

**Table 3:** Factors associated with dental caries experience (D_4_MFT ≥ 1)^a^ among adults in Butajira HDSS, South-central Ethiopia

Explanatory Variables		Unadjusted Model		Adjusted Model	
		PR^**f**^	95% CI	PR^**f**^	95% CI
Age group	18–24	1	[Reference]	1	[Reference]
	25–34	1.53**	1.11–2.10	1.28	0.95–1.71
	35–44	2.59***	1.92–3.48	1.76***	1.31–2.39
	45–54	2.64***	1.95–3.56	1.80***	1.33–2.43
	55–64	2.95***	2.21–3.95	1.75***	1.29–2.38
	65+	3.18***	2.39–4.22	2.02***	1.50–2.74
Sex	Female	1	[Reference]	1	[Reference]
	Male	1.01	0.89–1.15	1.09	0.94–1.25
Educational status	No formal education	1	[Reference]	1	[Reference]
	Primary school	0.62***	0.54–0.72	0.76***	0.66–0.89
	Secondary school	0.42***	0.32–0.56	0.59***	0.44–0.80
	Higher education	0.40***	0.27–0.60	0.55**	0.37–0.82
Location	Urban	1	[Reference]	1	[Reference]
	Rural	1.27***	1.12–1.45	0.97	0.85–1.10
Utilisation of dental care services	Never	1	[Reference]	1	[Reference]
	Ever	1.68***	1.51–1.87	1.51***	1.36–1.68
Frequency of tooth cleaning	Never/ Rarely	1	[Reference]	1	[Reference]
	Less than once a day	0.74***	0.63–0.87	1.01	0.78–2.60
	Once a day	0.61***	0.49–0.76	0.93	0.70–2.26
	Twice or more per day	0.87	0.73–1.04	1.11	0.86–1.44
Tooth cleaning material	Toothbrush	1	[Reference]	1	[Reference]
	Mefakiya^b^ / Chewstick	1.32*	1.16–2.79	0.97	0.70–1.36
	Wooden toothpicks	1.37**	1.21–3.31	0.94	0.66–1.33
	Other	1.46*	0.95–6.02	1.05	0.69–1.61
Use of toothpaste	Yes	1	[Reference]	1	[Reference]
	No	1.52***	1.22–1.89	0.92	0.63–1.34
Frequency of Khat chewing	Never	1	[Reference]	1	[Reference]
	Less than weekly	1.28**	1.08–3.12	0.96	0.80–1.15
	More than once a week	1.33***	1.30–3.31	1.16	0.98–1.36
	Every day/nearly every day	1.52***	2.01–6.30	1.38***	1.15–1.64
Level of sugar consumption risk	Higher^c^	1	[Reference]	1	[Reference]
	Moderate^d^	0.99	0.76–1.28	0.95	0.75–1.20
	Lower^e^	1.29*	1.02–1.65	1.12	0.90–1.40

^a^DT: ICDAS 4 and above, ^b^Mefakiya: an Amharic name for chewstick, ^c^More than once a week, ^d^Between once a month and once a week, ^e^Rarely or never, ^f^Poisson regression models with robust standard error were fitted, and prevalence ratios (PR) were reported

*p < 0.05; **p < 0.01; ***p < 0.001

## Discussion

In this cross-sectional study amongst a mixed rural and urban population of south-central Ethiopia, 6 out of 10 adults were found to have dental caries experience. This study is one of the few oral health research projects to have been carried out in the country and the first population-based oral health survey to be part of a large multi-disciplinary global health project providing an overview of adult oral health in the community and the most extensive in nature. This research has certain limitations which need to be acknowledged. First, it was conducted in a relatively ethnically homogenous population, which therefore cannot be considered nationally representative. Second, almost half (46.8%) of the study participants were from the urban area due to the accessibility challenge in some rural areas. The 10.8% non-response rate (129 persons out of the 1200 proposed sample size) is also attributed to the non-accessibility of some rural households. Since a large proportion (80.0%) of the general population in the country is rural, this again needs to be recognised as unrepresentative of the national situation; however, it allowed comparison between the rural and urban settings and given the study findings whereby the differences between urban and rural settings were not significantly different in the adjusted Poisson regression model, this may not be a major factor. Third, the people who stayed at home and were available to participate in the data collection might be different from those who were out engaging in work and other activities and thus possibly overrepresented. However, every effort was made to ensure that people who worked away from home were included in the study, although most men working in the fields might have been missed. Fourth, the questionnaire data were collected by face-to-face interview, which means participants may have given more socially desirable answers about their lifestyle and behaviours. Nonetheless, given the very low level of education in this community, this was the most appropriate survey method. Fifth, the sample size was calculated based on surgical care needs; however as surgical care needs are less common than the oral diseases, this provided a substantial sample for the oral health survey when compared with other national dental surveys [28], and our findings will provide a sound basis to inform future epidemiological studies in Ethiopia.

The magnitude of untreated obvious caries (clinically visible lesions representing demineralisation in the middle third of the dentine, and above indicated by the ICDAS 4 threshold) in the study participants was high; if the early and sub-clinical lesions were included, which would require optimal clinical and radiographic examinations [39], a higher value would result. These findings suggest an increasing burden of NCDs in general [40]. Interestingly, a higher proportion of dental caries experience was observed in rural areas, unlike a previous study in Ethiopia which reported a higher prevalence of caries in urban areas (61.1%) [13]. However, the probability of experiencing dental caries was almost the same for both locations in the adjusted Poisson regression model. Although rural areas are generally suggested to have less access to cariogenic foods, this may be changing with the nutrition transition; in addition to the limited access to dental care and preventive services as compared to urban areas [41]. Similar issues exist in both lower and higher income countries, whereby the rural population has more untreated caries [34, 41]. Furthermore, according to Ogunbodede et.al (2015), accessibility and utilisation of modern dental services is still a problem for the rural population in the African and middle east region, thus oral health inequalities are marked between rural and urban populations in the region [34].

In the study participants, the probability of experiencing caries increased with age and the pattern of the condition is also cumulative throughout life by its nature [33]. This finding mirrors the emerging result of the Dunedin longitudinal study (the 1972/3 birth cohort) of dental caries trajectory assessment in which the participant’s dental caries experience in the different categories of the trajectory increased with age [42]. However, the difference between males and females regarding their dental caries experience was not significant in our study, as in the recent global burden of oral health study findings [43, 44]. Previous studies elsewhere reported sex differences in the prevalence and severity of caries, which has been explained by a range of factors including dietary patterns [45–47].

The regression models in this study demonstrated the probability of experiencing dental caries significantly reduced with increasing educational attainment of adults. Education influences an individual’s position in the society, also determines their income and day-to-day activities. This in turn influences the risk of one’s exposure to different diseases [24], including dental caries [33]. As in other health outcomes, oral diseases in general are patterned across the social gradient, and they are directly influenced by the social determinants of health and inequalities in the society [48–50]. It might have been expected that in a low-income country, the converse would be seen where higher social classes have greater access to sugar.

Health behaviours, whilst important risk factors for oral disease [8], are less likely to be significant in our model. The association of oral hygiene practice and sugar consumption was no longer present after the statistical adjustment. However, people who reported cleaning their teeth twice or more per day were those with a higher dental caries experience. This might be explained by the fact that those who already have a disease problem tend to perceive the severity, so that they adapt healthy behaviours and clean their teeth more [51], but do not appear to be using fluoride toothpaste [52]. Most of our study participants practiced traditional way of tooth brushing using mefakiya (chewstick). Studies suggest that, traditional chewsticks are as effective as toothbrushes in mechanically removing tooth debris, and they are even better in some cases if used properly [53]. The chemical compositions in some of the chewsticks also inhibit plaque bacteria [53, 54]. However, their effectiveness compared with brushing with fluoridated and non-fluoridated toothpaste is not yet known [53]. Apart from the use of toothpaste, possibly fluoridated, there is no known fluoride supplement in our study area. Thus, it can be assumed that exposure to fluoride in the community is low. This can increase vulnerability of individuals to caries, as optimum and controlled use of fluoride is one of the best preventive methods for dental caries [55–57].

Amongst the health behaviours assessed in this survey, chewing Khat daily was associated with increased probability of dental caries experience when keeping other variables in the model constant. Khat chewing has been suggested to be associated with some oral conditions including periodontal diseases, attrition of tooth surface and tooth discolouration, oral cancer, and other mucosal diseases. According to Al-Maweri (2018), Khat itself is not cariogenic. However, it has a dehydrating effect on the oral mucosa and a bitter taste. Consequently, Khat chewers tend to consume a large quantity of non-alcoholic fluids, such as carbonated soft drinks, water, and coffee (usually sweetened). Some also supplement the leaves with refined sugar and it also increases the tendency to tobacco smoking [37]. A cohort study of 98 Yemeni Khat chewers and 101 non-chewers, aged 18–35 years with early occlusal caries suggested a higher risk of dental caries progression in low-income Khat chewers as compared to the non-chewers, although the study follow-up was short [58].

Whilst utilisation of dental care was generally uncommon, it was notable in those with dental caries experience in our study, only just over one-third reported utilising a dental care service. However, those who utilised the service had a higher probability of dental caries experience than those who never did. This might suggest that dental visit was perceived need driven and given the level of untreated diseases, care was probably limited to the offending lesion. In this regard, the main reasons for not utilising dental care services are generally multifactorial [38], and it can also be highlighted by the barriers of access related to the direct and indirect costs and the availability of the service. A study of Jordanian adults on factors influencing access to dental care also reported the cost of treatment and perceived lack of treatment need, following lack of time as main reasons for not utilising a dental care service [59]. However, our study participants did not highlight lack of time as a reason, but mainly distance to the dental service and cost of treatments. Another study among adult city-dwellers in Burkina Faso also highlighted cost of dental treatment as the main barrier to dental visits [60]. Dental treatment is known to expose households to unpredictable and significant financial costs; recent research has shown, the larger portion of the disposable income in low income households could be used for dental treatment mainly in out-of-pocket payment situations [61]. This could also be the case in Ethiopia, as out-of-pocket payment is the main method of funding healthcare [62].

According to our findings, half of the people with dental caries experience had missing teeth due to caries, and less than 1% had dental fillings. This indicates that tooth extraction is the most widely used treatment modality for dental caries in the area, which may be due to the limited access to care or choice of treatment option, as well as late presentation with disease. These can also be attributable to the significant proportion of reported PUFA and feeling of pain or problem as consequences of untreated caries. Besides, just over half of the people in this study reasoned ‘never experiencing any dental problem’ for not seeking dental care, despite the high prevalence of caries and report of experiencing pain or discomfort during the last year. Their reports seem to involve contradictory evidence. However, this could possibly indicate the low level of awareness about oral disease, specifically dental caries and their complications in the community. This in turn suggests the strong need of population centred health promotion programmes in the area, to facilitate the community having better awareness and control over their health outcomes, as to prevent disease and improve their health [63]. However, socioeconomic conditions can also determine access to preventive and restorative dental treatments [64]. Access to care can be improved by strengthening the health system in terms of expanding coverage, and providing good health service with optimum use of available resources, and implementing other relevant strategies [65]. The use of primary care services helped most industrialised countries to achieve greater equity so that people with greater health needs can get better access to services [66], albeit in response to symptoms and with less regularity. Reviews of oral health in the African region also suggested those challenges can be addressed by creating universal and equitable access to quality, affordable and appropriate oral health services [67].

In general, our findings demonstrated that health behaviours had less influence on oral health, apart from frequent Khat chewing and utilisation of care. However, sociodemographic conditions, particularly age and educational status and markedly affected the magnitude of untreated caries in our study area. Although not statistically significant, the prevalence of caries differed according to location of residence. This emphasises the role of social determinants in oral diseases and health outcomes in general, inferring a marked inequity in oral health and utilisation of care [66]. Despite being preventable and treatable, the higher prevalence of untreated caries, along with the very low utilisation of dental care services and inadequate oral health awareness in the society indicate the inevitable need of focusing on oral health promotion within the health system. However, accessibility of dental services and cost of treatment were identified as possible barriers to access to care in this study. Having an under-developed healthcare system, particularly in countries like Ethiopia, also affects oral diseases and uptake of care. Moreover, the pattern of services utilisation can influence the distribution of resources, development of strategic health policies and timely response by different social groups [36]. Therefore, an ideal oral healthcare system involving an emphasis on health promotion and disease prevention; integration with the rest of the healthcare system; universal, cost-effective, sustainable and equitable care is advocated to empower communities and individuals to create conditions conducive to health [68].

This research provides important insights to dental caries experience and access to care in Ethiopia, providing pilot data to inform a nationally representative survey of oral health. This can be an input towards planning appropriate workforce capacity building through modelling future scenarios for dental service provision and expand healthcare coverage. It will also inform the future general and oral health policy and strategy as to improve the population health and redirect the priority towards oral health. Furthermore, it will be important to develop research informing feasible ways of addressing oral healthcare needs of urban and rural populations; inform policy and strategy on health promotion and healthcare services. Further research should also investigate oral health in relation to NCDs, together with the possible relationship of Khat and dental caries.

## Conclusion

The finding suggests that there is a high burden of dental caries, considerable consequences of untreated disease and low uptake of care amongst adults in the district of Butajira. It also implies the influence of sociodemographic conditions on their health outcome and marked social inequity. Caries experience increases with age and is less prevalent amongst educated and urban populations. However, it is not largely influenced by health behaviours. Oral health awareness is low and there is a high need for dental care in general. Overall, this indicates a clear need of health system strengthening interventions with a focus on oral health promotion and better access to care.

## Supplementary Information

**Additional file 1.** Oral Health Questionnaire for Adults. Questionnaire utilised for the dental/surgical survey within in the ASSET project in Ethiopia.

## Data Availability

The datasets used and/or analysed during the current study are available from the corresponding author on reasonable request.

## Abbreviations

ASSET: Health System Strengthening in sub-Saharan Africa
DMFT: Decayed, Missed and Filled Teeth
HDSS: Health and Demographic Surveillance Site
ICCMS: International Caries Classification and Management System
ICDAS: International Caries Detection and Assessment System
NCD: Non-Communicable Diseases
NIHR: National Institute for Health Research
ODK: Open Data Kit
PR: Prevalence Ratio
PUFA: Pulpal involvement, Ulcer, Fistula and Abscess
SNNPR: Southern Nations, Nationalities, and Peoples’ Region
WHO: World Health Organization

## References

[1] Glick M, Williams DM, Kleinman DV, Vujicic M, Watt RG, Weyant RJ (2016). A new definition for oral health developed by the FDI world dental federation opens the door to a universal definition of oral health. J Am Dent Assoc.

[2] Peres MA, Macpherson LMD, Weyant RJ, Daly B, Venturelli R, Mathur MR (2019). Oral diseases: a global public health challenge. Lancet.

[3] Petersen PE, Kwan S (2011). Equity, social determinants and public health programmes--the case of oral health. Community Dent Oral Epidemiol.

[4] Marcenes W, Kassebaum NJ, Bernabe E, Flaxman A, Naghavi M, Lopez A (2013). Global burden of oral conditions in 1990-2010: a systematic analysis. J Dent Res.

[5] Jin LJ, Lamster IB, Greenspan JS, Pitts NB, Scully C, Warnakulasuriya S (2016). Global burden of oral diseases: emerging concepts, management and interplay with systemic health. Oral Dis.

[6] Gouda HN, Charlson F, Sorsdahl K, Ahmadzada S, Ferrari AJ, Erskine H (2019). Burden of non-communicable diseases in sub-Saharan Africa, 1990-2017: results from the global burden of disease study 2017. Lancet Glob Health.

[7] Kassebaum NJ, Bernabe E, Dahiya M, Bhandari B, Murray CJ, Marcenes W (2014). Global burden of severe periodontitis in 1990-2010: a systematic review and meta-regression. J Dent Res.

[8] Abid A, Maatouk F, Berrezouga L, Azodo C, Uti O, El-Shamy H (2015). Prevalence and severity of Oral diseases in the Africa and Middle East region. Adv Dent Res.

[9] International Monetary Fund (2019). World economic outlook.

[10] Central Intelligence Agency. The World Factbook. Ethiopia; 2020. 17 March 2020. Available from: https://www.cia.gov/library/publications/the-world-factbook/geos/et.html

[11] Mulu W, Demilie T, Yimer M, Meshesha K, Abera B (2014). Dental caries and associated factors among primary school children in Bahir Dar city: a cross-sectional study. BMC Res Notes.

[12] Ayele FA, Taye BW, Ayele TA, Gelaye KA (2013). Predictors of dental caries among children 7-14 years old in Northwest Ethiopia: a community based cross-sectional study. BMC Oral Health.

[13] Tafere Y, Chanie S, Dessie T, Gedamu H (2018). Assessment of prevalence of dental caries and the associated factors among patients attending dental clinic in Debre Tabor general hospital: a hospital-based cross-sectional study. BMC Oral Health.

[14] Burnett D, Aronson J, Asgary R (2016). Oral health status, knowledge, attitudes and behaviours among marginalized children in Addis Ababa, Ethiopa. J Child Health Care.

[15] United Nations (2020). Sustainable Development Goals.

[16] Guideline for implementation of a patient referral system (2010). Medical Services Directorate.

[17] Bradley E, Hartwig KA, Rowe LA, Cherlin EJ, Pashman J, Wong R (2008). Hospital quality improvement in Ethiopia: a partnership-mentoring model. Int J Qual Health Care.

[18] Prasad M, Manjunath C, Murthy AK, Sampath A, Jaiswal S, Mohapatra A (2019). Integration of oral health into primary health care: a systematic review. J Family Med Prim Care.

[19] Petersen PE (2014). Strengthening of oral health systems: oral health through primary health care. Med Princ Pract.

[20] Gallagher JE, Hutchinson L (2018). Analysis of human resources for oral health globally: inequitable distribution. Int Dent J.

[21] World Health Organization (2019). Oral Health Manpower.

[22] Gile PP, Buljac-Samardzic M, Klundert JV (2018). The effect of human resource management on performance in hospitals in sub-Saharan Africa: a systematic literature review. Hum Resour Health.

[23] Hagopian A, Thompson MJ, Fordyce M, Johnson KE, Hart LG (2004). The migration of physicians from sub-Saharan Africa to the United States of America: measures of the African brain drain. Hum Res Health.

[24] Dahlgren G, Whitehead M (1991). Policies and strategies to promote social equity in health.

[25] Baru A, Murugan P (2016). Social determinants of vulnerability to ill-health: evidences from Mendi town, Western Ethiopia. J Health Soc Sci.

[26] Berhane Y, Wall S, Kebede D, Emmelin A, Enqueselassie F, Byass P, et al. Establishing an epidemiologic al field laboratory in rural areas - potentials for public health research and interventions the Butajira rural health Programme 1987-1999. Ethiop J Health Dev. 1999;13.

[27] World Health Organization (2013). Oral health surveys: basic methods.

[28] O’Sullivan I, Lader D, Beavan-Seymour C, Chenery V, Fuller E, Sadler K (2011). Foundation Report: Adult Dental Health Survey 2009 (Technical information).

[29] Criteria Manual - International Caries Detection and Assessment System (ICDAS II). Revised in December and July 2009 (Bogota, Colombia and Budapest, Hungary) – from a Workshop held in Baltimore, Maryland, March 12th–14th, 2005: ICDAS Foundation; 2009. Available from: http://www.icdas.org

[30] Abebe W (2018). Khat and synthetic cathinones: emerging drugs of abuse with dental implications. Oral Surg Oral Med Oral Pathol Oral Radiol.

[31] Gebissa E (2010). Khat in the horn of Africa: historical perspectives and current trends. J Ethnopharmacol.

[32] Monse B, Heinrich-Weltzien R, Benzian H, Holmgren C, van Palenstein Helderman W (2010). PUFA--an index of clinical consequences of untreated dental caries. Community Dent Oral Epidemiol.

[33] Costa SM, Martins CC, Bonfim Mde L, Zina LG, Paiva SM, Pordeus IA (2012). A systematic review of socioeconomic indicators and dental caries in adults. Int J Environ Res Public Health.

[34] Ogunbodede EO, Kida IA, Madjapa HS, Amedari M, Ehizele A, Mutave R (2015). Oral health inequalities between rural and urban populations of the African and Middle East region. Adv Dent Res.

[35] Griffin SO, Regnier E, Griffin PM, Huntley V (2007). Effectiveness of fluoride in preventing caries in adults. J Dent Res.

[36] Chidzonga MM, Carneiro LC, Kalyanyama BM, Kwamin F, Oginni FO (2015). Determinants of Oral diseases in the African and Middle East region. Adv Dent Res.

[37] Al-Maweri SA, Warnakulasuriya S, Samran A. Khat (*Catha edulis*) and its oral health effects: An updated review. J Investig Clin Dent. 2018;9(1). 10.1111/jicd.12288. Epub 2017 Aug 19. PMID: 28834423. 10.1111/jicd.12288 28834423

[38] Harris RV, Pennington A, Whitehead M (2017). Preventive dental visiting: a critical interpretive synthesis of theory explaining how inequalities arise. Community Dent Oral Epidemiol.

[39] Ismail AI, Pitts NB, Tellez M, Banerjee A, Authors of International Caries C, Management S (2015). The International Caries Classification and Management System (ICCMS) An Example of a Caries Management Pathway. BMC Oral Health.

[40] Melaku YA, Temesgen AM, Deribew A, Tessema GA, Deribe K, Sahle BW (2016). The impact of dietary risk factors on the burden of non-communicable diseases in Ethiopia: findings from the global burden of disease study 2013. Int J Behav Nutr Phys Act.

[41] Fos P, Hutchison L (2003). The state of rural Oral health: a literature review -. Rural healthy people 2010: a companion document to healthy people 2010.

[42] Broadbent JM, Thomson WM, Poulton R (2008). Trajectory patterns of dental caries experience in the permanent dentition to the fourth decade of life. J Dent Res.

[43] Kassebaum NJ, Bernabe E, Dahiya M, Bhandari B, Murray CJ, Marcenes W (2015). Global burden of untreated caries: a systematic review and metaregression. J Dent Res.

[44] Kassebaum NJ, Smith AGC, Bernabe E, Fleming TD, Reynolds AE, Vos T (2017). Global, regional, and National Prevalence, incidence, and disability-adjusted life years for Oral conditions for 195 countries, 1990-2015: a systematic analysis for the global burden of diseases, injuries, and risk factors. J Dent Res.

[45] Shaffer JR, Wang X, McNeil DW, Weyant RJ, Crout R, Marazita ML (2015). Genetic susceptibility to dental caries differs between the sexes: a family-based study. Caries Res.

[46] Ferraro M, Vieira AR (2010). Explaining gender differences in caries: a multifactorial approach to a multifactorial disease. Int J Dent.

[47] Lukacs JR, Largaespada LL (2006). Explaining sex differences in dental caries prevalence: saliva, hormones, and “life-history” etiologies. Am J Hum Biol.

[48] Watt R, Sheiham A (1999). Inequalities in oral health: a review of the evidence and recommendations for action. Br Dent J.

[49] Marmot M (2017). Social justice, epidemiology and health inequalities. Eur J Epidemiol.

[50] Moyses SJ (2012). Inequalities in oral health and oral health promotion. Braz Oral Res.

[51] Kasmaei P, Amin Shokravi F, Hidarnia A, Hajizadeh E, Atrkar-Roushan Z, Karimzadeh Shirazi K (2014). Brushing behavior among young adolescents: does perceived severity matter. BMC Public Health.

[52] Public Health England. In: Health, editor. Delivering better oral health: an evidence-based toolkit for prevention. 3rd ed. London: Public Health England (PHE); 2017.

[53] Dahiya P, Kamal R, Luthra RP, Mishra R, Saini G (2012). Miswak: a periodontist's perspective. J Ayurveda Integr Med.

[54] Olsson B (1978). Efficiency of traditional chewing sticks in oral hygiene programs among Ethiopian schoolchildren. Community Dent Oral Epidemiol.

[55] Petersen PE, Ogawa H (2016). Prevention of dental caries through the use of fluoride--the WHO approach. Community Dent Health.

[56] Horst JA, Tanzer JM, Milgrom PM (2018). Fluorides and other preventive strategies for tooth decay. Dent Clin N Am.

[57] Whelton HP, Spencer AJ, Do LG, Rugg-Gunn AJ (2019). Fluoride revolution and dental caries: evolution of policies for global use. J Dent Res.

[58] Al-Alimi KR, Razak AAA, Saub R (2018). Is Khat chewing habit a risk factor for occlusal caries progression?. Afr Health Sci.

[59] Obeidat SR, Alsa'di AG, Taani DS (2014). Factors influencing dental care access in Jordanian adults. BMC Oral Health.

[60] Varenne B, Petersen PE, Fournet F, Msellati P, Gary J, Ouattara S (2006). Illness-related behaviour and utilization of oral health services among adult city-dwellers in Burkina Faso: evidence from a household survey. BMC Health Serv Res.

[61] Bernabe E, Masood M, Vujicic M (2017). The impact of out-of-pocket payments for dental care on household finances in low and middle income countries. BMC Public Health.

[62] Tolla MT, Norheim OF, Verguet S, Bekele A, Amenu K, Abdisa SG (2017). Out-of-pocket expenditures for prevention and treatment of cardiovascular disease in general and specialised cardiac hospitals in Addis Ababa, Ethiopia: a cross-sectional cohort study. BMJ Glob Health.

[63] World Health Organization (1986). Ottawa Charter for Health Promotion.

[64] Cheema J, Sabbah W (2016). Inequalities in preventive and restorative dental services in England, Wales and Northern Ireland. Br Dent J.

[65] World Health Organization (2007). Everybody business: strengthening health systems to improve health outcomes.

[66] Starfield B (2011). The hidden inequity in health care. Int J Equity Health.

[67] World Health Organization. Regional Office for Africa. Promoting Oral Health in Africa: Prevention and control of oral diseases and noma as part of essential noncommunicable disease interventions. World Health Organization. Regional Office for Africa. 2016. https://apps.who.int/iris/handle/10665/205886.

[68] Tomar SL, Cohen LK (2010). Attributes of an ideal oral health care system. J Public Health Dent.

