# Supplementary material for: Dental caries experience and associated factors in adults: a cross-sectional community survey within Ethiopia
Source: BMC Public Health. 2021 Jan 21;21:180. doi: 10.1186/s12889-021-10199-9 (PMC7819221; doi:10.1186/s12889-021-10199-9)
Supplement: Supplementary file 1 — Additional file 1. Oral Health Questionnaire for Adults. Questionnaire utilised for the dental/surgical survey within in the ASSET project in Ethiopia. [file 12889_2021_10199_MOESM1_ESM.docx]

**Oral Health Questionnaire for Adults**

***Code no.: ______________ Date: __________________ Area Code: ______________***

***Recorder: _______________________ Dentist: ____________________________***

| ***Note*** | ***First, we would like you to answer some questions concerning your teeth*** |
| --- | --- |
| 1 | **How often during the past 12 months did you have toothache or feel discomfort due to your teeth?**   \| 1 \| Often \|  \| \| --- \| --- \| --- \| \| 2 \| Occasionally \|  \| \| 3 \| Rarely \|  \| \| 4 \| Never \|  \| \| 5 \| Don’t know \|  \| |
| 2 | **How would you describe the health of your teeth and gums?**   \|  \|  \| 1. **Teeth** \| 1. **Gums** \| \| --- \| --- \| --- \| --- \| \| 1 \| Excellent \|  \|  \| \| 2 \| Very Good \|  \|  \| \| 3 \| Good \|  \|  \| \| 4 \| Average \|  \|  \| \| 5 \| Poor \|  \|  \| \| 6 \| Very poor \|  \|  \| \| 9 \| Don’t know \|  \|  \| |
|  | ***Now, we would like you to answer some questions concerning the care of your teeth*** |
| 3 | **How often do you clean your teeth?**   \| 1 \| More than three times a day \|  \| \| --- \| --- \| --- \| \| 2 \| Twice or more a day \|  \| \| 3 \| Once a day \|  \| \| 4 \| Less than once a day \|  \| \| 5 \| Never/ rarely \|  \| |
| 4 | **Which of the following do you use to clean your teeth?**   \| 1 \| Toothbrush \|  \| \| --- \| --- \| --- \| \| 2 \| Wooden toothpicks \|  \| \| 3 \| Plastic toothpicks \|  \| \| 4 \| Thread (dental floss) \|  \| \| 5 \| Charcoal \|  \| \| 6 \| Mefakiya/ Chewstick \|  \| \| 7 \| Other (specify) \|  \| |
| 5 | \| **Do you use toothpaste to clean your teeth?** \| **Yes** \| **No** \| \| --- \| --- \| --- \| \|  \|  \| |
| 6 | **How long is it since you last saw a dentist (if ever)?**   \| 1 \| Less than 6 months \|  \| \| --- \| --- \| --- \| \| 2 \| 6-12 months \|  \| \| 3 \| More than one year (but less than 2 years) \|  \| \| 4 \| 2 years or more (but less than 5 years) \|  \| \| 5 \| 5 years or more \|  \| \| 6 \| Never received dental care/visited a dentist \|  \| |
|  | *If you did see a dentist during the last 12 months go to the next question*  *If you did not see a dentist during the last 12 months, go on to question 8* |
| 7 | **What was the reason of your last visit to the dentist? (Tick all that applies)**   \| 1 \| Consultation/advice \|  \| \| --- \| --- \| --- \| \| 2 \| Pain or trouble with teeth, gums, or mouth \|  \| \| 3 \| Treatment/follow-up treatment \|  \| \| 4 \| Routine check-up of teeth/treatment \|  \| \| 5 \| Don’t know/don’t remember \|  \| |
| 8 | **If you have never visited a dentist, what was the reason?** **(Tick all that applies)**   \| 1 \| Distance to the dental clinic \|  \| \| --- \| --- \| --- \| \| 2 \| Unfriendly dental worker \|  \| \| 3 \| Fear of losing a tooth \|  \| \| 4 \| Fear of painful treatment \|  \| \| 5 \| Long waiting time \|  \| \| 6 \| Cost of treatment \|  \| \| 7 \| I have never had any dental problem \|  \| |
| 9 | **Because of the state of your teeth or mouth, how often have you experienced any of the following problems during the past 12 months?**   \|  \| Very often**^4^** \| Fairly Often**^3^** \| Sometimes**^2^** \| No**^1^** \| Don’t know**^0^** \| \| --- \| --- \| --- \| --- \| --- \| --- \| \| Difficulty in biting foods \|  \|  \|  \|  \|  \| \| Difficulty chewing foods \|  \|  \|  \|  \|  \| \| Difficulty with speech/trouble pronouncing words \|  \|  \|  \|  \|  \| \| Dry mouth \|  \|  \|  \|  \|  \| \| Felt embarrassed due to appearance of teeth \|  \|  \|  \|  \|  \| \| Felt tense because of problems with teeth or mouth \|  \|  \|  \|  \|  \| \| Have avoided smiling \|  \|  \|  \|  \|  \| \| Had sleep that is often interrupted \|  \|  \|  \|  \|  \| \| Have taken days off work \|  \|  \|  \|  \|  \| \| Difficulty doing usual activities \|  \|  \|  \|  \|  \| \| Felt less tolerant of spouse or people who are close to you \|  \|  \|  \|  \|  \| \| Have reduced participation in social activities \|  \|  \|  \|  \|  \| |
| 10 | **How often do you use any of the following types of tobacco?**   \|  \| Every day**^6^** \| Several times a week**^5^** \| Once a week**^4^** \| Several times a month**^3^** \| Seldom**^2^** \| Never**^1^** \| \| --- \| --- \| --- \| --- \| --- \| --- \| --- \| \| Cigarettes \|  \|  \|  \|  \|  \|  \| \| Pipe \|  \|  \|  \|  \|  \|  \| \| Cigars \|  \|  \|  \|  \|  \|  \| \| Chewing tobacco \|  \|  \|  \|  \|  \|  \| \| Other \|  \|  \|  \|  \|  \|  \| |
| 11 | \| How frequently do you chew Khat? \| Every day or nearly everyday**^6^** \| Once or twice a week**^5^** \| 1 to 3 times per month**^4^** \| Less than monthly**^3^** \| Not in the last year**^2^** \| Never**^1^** \| \| --- \| --- \| --- \| --- \| --- \| --- \| --- \| \|  \|  \|  \|  \|  \|  \| |
| 12 | **How often do you eat or drink any of the following foods/ drinks?**   \|  \| Four times a day**^6^** \| Three times a day**^5^** \| Two times a day**^4^** \| Once a day**^3^** \| Less than once a day**^2^** \| Rarely/ Never**^1^** \| \| --- \| --- \| --- \| --- \| --- \| --- \| --- \| \| Fruits \|  \|  \|  \|  \|  \|  \| \| Cakes or biscuits \|  \|  \|  \|  \|  \|  \| \| Candy or chocolates \|  \|  \|  \|  \|  \|  \| \| Soft drinks like coca cola \|  \|  \|  \|  \|  \|  \| \| Fruit juices \|  \|  \|  \|  \|  \|  \| \| Tea, coffee or milk with sugar \|  \|  \|  \|  \|  \|  \| \| Other, specify \|  \|  \|  \|  \|  \|  \| |
| 13 | **Finally, is there anything else you would like to say about your dental health or about dentists in general?** |
